# Study on the effectiveness of modified colonoscopy nursing pads in colonoscopy

**DOI:** 10.1186/s12876-022-02493-6

**Published:** 2022-12-20

**Authors:** Zhenfei Bao, Chunyan Hu, Lei Zhu, Weihong Wang

**Affiliations:** Department of Endoscopy Center, Ningbo First Hospital, No.59 of Liuting Street, Haishu District, Zhejiang, 315010 China

**Keywords:** Colonoscopy, Nursing pad, Modification, Postural guidance, Clinical effect

## Abstract

**Objective:**

To investigate the effect of modified colonoscopy nursing pads in colonoscopy.

**Methods:**

A total of 262 subjects who underwent colonoscopy at our endoscopy center between September 1, 2021 and February 28, 2022 were selected and randomly divided into a control group and an experimental group, with 131 cases in each group. The control group used conventional nursing pads, while the experimental group used modified nursing pads. The success rate of the first correct position, the time spent by the nurse to guide the correct position, the bed unit contamination rate, the contamination rate of the operator's protective equipment, the privacy protection of the examinees and the satisfaction degree after the examination were compared between the two groups.

**Results:**

The success rate of the first correct position of the examinees in the experimental group was significantly higher than that of the control group (*P* < 0.05), and the time spent by the nurses to guide the correct position in the experimental group was less than that of the control group (*P* < 0.05). The bed unit contamination rate and operator's protective equipment contamination rate of the experimental group were lower than those of the control group, and the satisfaction degree of the examinees was higher in the experimental group than in the control group, and the differences were statistically significant (*P* < 0.05).

**Conclusion:**

The modified colonoscopy nursing pad can save the time of correct colonoscopy positioning of examinees, improve the efficiency of colonoscopy, reduce the workload of nursing staff, effectively protect the privacy of patients, reduce the bed unit contamination and protective equipment contamination, and then improve the comfort and satisfaction of patients.

**Supplementary Information:**

The online version contains supplementary material available at 10.1186/s12876-022-02493-6.

## Introduction

Among the specialized examinations in gastroenterology, colonoscopy is the simplest, safest and most effective method to detect intestinal tumors and precancerous lesions [1]. During the colonoscopy, the position of the patient has a significant impact on the success rate of colonoscopy, the detection rate of lesions and the patient's examination experience [2–4]. In order to improve the success rate of colonoscopy, the patient is usually required to adopt the left lateral position, which requires the nursing staff to provide guidance and education on the position of the patient [5, 6]. Due to age, language, and literacy differences as well as doctor-patient communication difficulty, many examinees often need to spend too much time to try repeatedly until the position is correct, and nurses will also give repeated instructions on the position, which directly leads to the reduction of examination efficiency. In addition, the unconscious discharge of fecal sewage during the examination is likely to contaminate the bed unit and the protective equipment of the operator, causing a certain burden on the maintenance of the endoscopy room environment. The examinee may feel nervous and anxious because of the exposure of private parts, which directly affects the examinee's examination experience. Therefore, our center made a modified colonoscopy nursing pad, and achieved good results in the application of colonoscopy, which is reported below.

## Data and methods

### Clinical information

A total of 262 subjects who underwent colonoscopy in our endoscopy center between September 1, 2021 and February 28, 2022 were selected, and randomly divided into 131 cases in the control group and 131 cases in the experimental group according to the envelope method by performing informed consent procedures. The basic information such as gender, age, height, weight and education level of the subjects in both groups were collected and used for baseline characteristics analysis. This study was reviewed and approved by the ethics committee of our hospital.

### Inclusion and exclusion criteria

Inclusion criteria: (1) outpatients and inpatients who required colonoscopy or endoscopic treatment; (2) patients aged 18–75 years old; (3) patients who voluntarily participated in this study and signed the informed consent form. Exclusion criteria: (1) patients with previous history of colonoscopy; (2) patients who underwent painless gastrointestinal endoscopy; (3) patients who underwent examination through colostomy (artificial anus) after colon surgery; (4) patients with mental illness or dysgnosia; (5) patients who were unconscious; (6) patients with limb movement disorder and required assistance to position themselves.

### Production of modified nursing pads

**Fig. 1:**
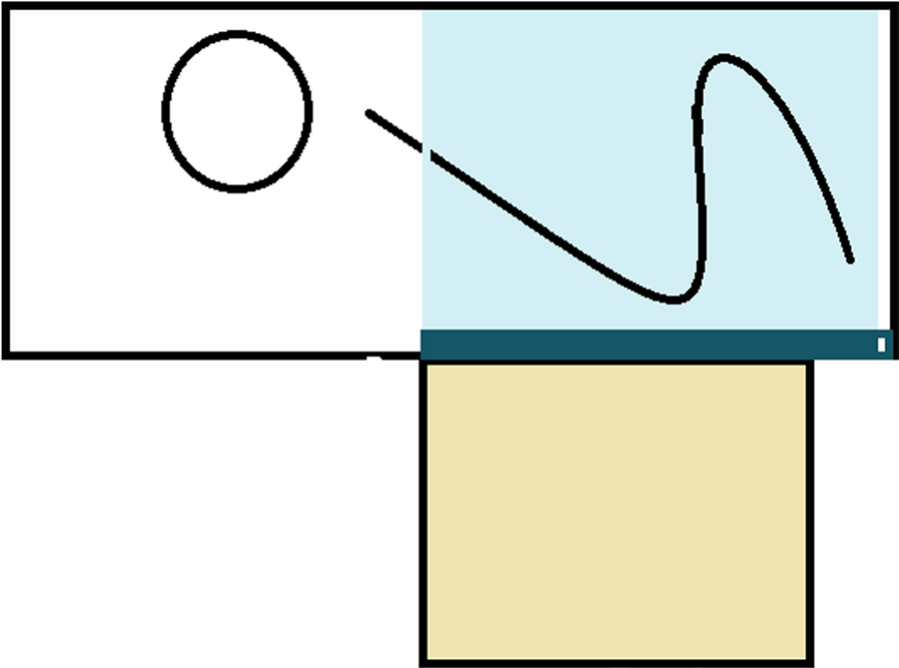
Schematic diagram of the modified colonoscopy nursing pad

The modified nursing pad (see Fig. 1) consisted of a 150 × 80 cm rectangular nursing pad main body and a 60 × 60 cm square nursing pad extension. The main body of the pad was drawn with a schematic diagram of the human lateral position by a surgical marker, so that the subject could intuitively understand how to place the body; the extension was designed in the middle and lower side of the main body and connected to the main part of the hip area, which could cover the subject's hip to abdomen laterally.

### Implementation method

In the control group, ordinary nursing pads, i.e. 150 × 80 cm nursing pads, were used. The nurse gave the regular instruction on position according to the three basic statements: "Please take off your shoes, please lie on your left side, please remove your pants to the root of your thighs", and might add verbal and body language instruction until the subject was in the correct position. The subject's buttocks were covered with a re-sterilized cloth sheet and toilet paper was provided to wipe the perianal area at the end of the procedure.

In the experimental group, homemade modified nursing pads were used, and the three basic statements of "please take off your shoes, please lie on your side according to the diagram on the nursing pads, please remove your pants to the root of your thighs" were used to instruct the subject on position during colonoscopy, and other verbal and body language instructions could be added until the subject was in the correct position, and the extended side of the modified nursing pad was used to cover the subject's buttocks. At the end of the procedure, the subject was instructed to wipe the perianal area with a buttock cover sheet and finally discard the entire used nursing pad after wrapping it.

### Evaluation indicators

#### The success rate of the subject being able to place the correct position on the first try

The correct position was the conventional initial position for colonoscopy, i.e., lying with both knees flexed in the left lateral position.

#### The time required for the nurse to instruct correct positioning

The timing started from the time the subject removed his shoes and ended until the patient was positioned correctly.

#### Bed unit and operator protective equipment contamination rate

During the operation, the bed unit and operator isolation clothing and waterproof shoes stained with fecal sewage visible to the naked eye were considered contamination.

#### Satisfaction degree of the examinees after the examination

The visual rating method was used to survey the satisfaction of the examinees for this colonoscopy. The questionnaire included 4 dimensions: environmental, physical, psychological, and spiritual, with a total of 10 items. A score of 0–10 was used, with 0 being very dissatisfied and 10 being very satisfied, with higher scores indicating higher satisfaction, and the privacy protection evaluation of the examinees was included in the satisfaction questionnaire (see Additional file 1).

### Statistical analysis

SPSS 22.0 was used for data collation and statistical analysis. The statistical data were expressed as n (%), and the chi-square test and Fisher's exact test were used to test between the groups; the measurement data were normally distributed with equal variance and expressed as mean ± SD, and the comparison of parameters between two groups was done by independent samples t-test; the measurement data did not conform to normal distribution were expressed as M(Q1, Q3), and the comparison between two groups was done by Wilcoxon rank sum test. *P* < 0.05 indicates that the difference was statistically significant.

## Results

### Analysis of baseline characteristics of examinees

The basic information such as gender, age, height, weight and education level of the subjects in both groups were collected and analyzed, and the results are shown in Table 1. The mean age of the subjects in the control group was (51.0 ± 15.9) and the mean age of the subjects in the experimental group was (52.3 ± 13.7), and the difference in age between the two groups was not statistically significant (*P* > 0.05). After statistical analysis, there was no statistically significant difference between the two groups of subjects in terms of gender ratio, height, weight, and education level (all *P* > 0.05), which indicated that the two groups of subjects were comparable.

**Table 1:** Baseline characteristics of patients

Factors	Experimental group (*n* = 131)	Control group (*n* = 131)	Total (*n* = 262)	*P*-value
Age, mean (SD), years old	52.3 ± 13.7	51.0 ± 15.9	51.6 ± 14.8	0.480^§^
*Gender, case. (%)*				
Male	72 (54.9)	69 (52.6)	141 (53.8)	0.710^‡^
Female	59 (45.0)	62 (47.3)	121 (46.1)	
Median height, (IQR,), cm	166.0 (160.0–172.0)	165.0 (158.0–170.0)	166.0 (160.0–172.0)	0.423*
Median weight, (IQR), kg	62.5 (56.0–73.0)	62.5 (55.5–72.0)	62.5 (56.0–73.0)	0.892*
*Education level, case (%)*				0.108^‡^
Illiterate and elementary school	36 (27.4)	45 (34.4)	81 (30.9)	
Secondary Schools	53 (40.4)	55 (41.9)	108 (41.2)	
University and above	42 (32.0)	31 (23.6)	73 (27.9)	

Mean, average; SD, standard deviation; IQR, interquartile range

*Wilcoxon rank sum test

^§^Two-sample *t* test, two independent samples *t* test

^‡^Chi-square test

### Comparison of the main outcome indicators between the two groups of examinees

In this study, the main outcome indicators were compared between the two groups of subjects. The time taken for correct positioning was 39.3 (30.4–48.7) s in the control group and 26.9 (21.6–38.7) s in the experimental group, which was significantly longer in the control group than in the experimental group (*P* < 0.05). In line with the comparison of the time to position, the nurse-guided positioning time in the experimental group was shorter than that in the control group, and the difference was statistically significant (*P* < 0.05). Specific analysis is shown in Table 2.

**Table 2:** Main outcome indicators

Factors	Experimental group	Control group	Total	*P*-value
Median positioning time, (IQR), seconds	26.9 (21.6–38.7)	39.3 (30.4–48.7)	33.0 (24.8–44.0)	0.000*
Median nurse-guided positioning time, (IQR), seconds	28.3 (21.5–36.3)	37.0 (29.0–46.6)	32.5 (24.5–42.0)	0.000*

IQR, interquartile range

*Wilcoxon rank sum test

### Comparison of secondary outcome indicators between the two groups of examinees

Among the secondary outcome indicators of the subjects in both groups (Table 3), 110 cases (83.9%) were correctly positioned at one time in the control group and 125 cases (95.4%) were correctly positioned at one time in the experimental group, and the success rate of correctly positioned at one time in the experimental group was higher than that in the control group, and the difference was statistically significant (*P* < 0.05). Compared with the control group, the nurses in the experimental group used less verbal guidance and even did not use body language for guidance. During the colonoscopy, there were 2 cases (1.5%) of clothing contamination, 20 cases (15.2%) of bed linen contamination and 3 cases (2.2%) of consultation room floor contamination in the control group, while no clothing, bed linen or consultation room floor contamination occurred in the experimental group; the bed unit contamination rate in the experimental group was significantly lower than that in the control group (*P* < 0.05). In 2021, the annual number of colonoscopy diagnosis and treatment in the endoscopy center of our hospital was 22,557 cases, and the pad single contamination rate in the control group was 15.2%. In theory, the expenditure of the control group should be increased by 22,557*15.2%*3.78 = 12,960.35 yuan. The analysis of the satisfaction degree of examinees after examination showed that the post-examination satisfaction degree of examinees in the experimental group was significantly higher than that in the control group, and the difference was statistically significant (*P* < 0.05).

**Table 3:** Secondary outcome indicators

Factors	Experimental group (*n* = 131)	Control group (*n* = 131)	Total (*n* = 262)	*P*-value
correctly positioned at one time, case. (%)	125 (95.4)	110 (83.9)	235 (89.7)	0.002^‡^
Number of nurse instructional statements greater than 6, case. (%)	1 (0.7)	16 (12.2)	17 (6.5)	0.000^a^
Use of body language, case. (%)	0 (0.0)	2 (1.5)	2 (0.7)	0.498^a^
Patient clothing contamination, case. (%)	0 (0.0)	2 (1.5)	2 (0.7)	0.498^a^
Bed linen contamination, case. (%)	0 (0.0)	20 (15.2)	20 (7.6)	0.000^a^
Consultation room floor contamination, case. (%)	0 (0.0)	3 (2.2)	3 (1.1)	0.247^a^
Patient satisfaction degree, mean (SD), score	99.8 ± 0.7	98.7 ± 2.0	99.24 ± 1.6	0.000

^a^Two-tailed Fisher exact test, Fisher exact probability method

^‡^Chi-square test

## Discussion

Intestinal endoscopy provides a specific morphological description of upper intestinal pathology and plays an important role in the diagnosis of intestinal tumors and precancerous lesions [7]. The success rate of colonoscopy is related to various factors such as the initial operation performed by the operator during insertion [8] and the psychological and physiological sedation level of the subject [9]. Correct positioning of the subject before the start of colonoscopy can effectively improve the success rate of colonoscopy [5, 10]. In recent years, some researchers have designed nursing pads with special functions such as pressure sore prevention, herbal healing, and physical cooling [11–13]. However, few nursing tools have been reported in the literature to assist in positioning during colonoscopy. In this study, the homemade modified colonoscopy nursing pad also has a diagram of human lateral position, which makes it easy for the examinee to understand the position at a glance and instructs the examinee how to place the position visually, even for the examinee with low literacy and communication difficulties. In this study, the use of homemade modified colonoscopy nursing pads significantly shortened the time for correct positioning of the examinee (*P* < 0.05), eliminated the need for excessive verbal and body language instruction by nursing staff, and reduced the workload of medical and nursing staff in teaching colonoscopy positions, thus improving the overall work efficiency.

Many hospitals usually hang colonoscopy posture demonstration diagrams on the walls of the consultation room, or play pre-colonoscopy posture instructional videos on a loop to guide the examinees on how to properly posture. However, some examinees were unable to correctly position themselves at once or quickly when faced with a blank nursing pad. This requires verbal or body language instruction by nursing staff before the examination, which greatly reduces the efficiency of colonoscopy. Chen Jia et al. designed a colonoscopy position marker sheet to improve the efficiency of colonoscopy patient positioning and save nurses' instruction time before operation [14]. Many patients are reluctant to undergo colonoscopy, partly because the exposure of private areas tends to cause embarrassment to patients. The colonoscopy marker bed sheet designed by Chen Jia et al. [14] only serves as a postural guide and does not protect the privacy of the examinee, which is lacking in humanized care. During colonoscopy, repeatedly washed and disinfected sheets are usually used to cover the buttocks of the patient in order to protect the privacy of the patient. If the cloth sheets are not repeatedly cleaned and disinfected for use and changed by each person, a large number of cloth sheets need to be equipped, which increases the expenditure on cleaning and disinfection and raises the cost of the endoscopy room, and there is a risk of cross infection if the cloth sheets cannot be changed by each person [15]. In addition, subjects usually defecate involuntarily during the examination, leading to contamination of the subject's clothing, bed unit and endoscopy room environment, which affects the patient's examination experience. In this study, the side extensions of the homemade modified colonoscopy pads were seamlessly connected with the main part of the pads and cover the buttocks of the examinees, which protected their privacy, satisfied the need of the examinees to be respected, and relieved the tension and anxiety of the examinees due to the exposure of their private parts; the pads were used once and changed once, which meets the requirements of the hospital, and can reduce the contamination rate of the bed unit, thus reducing the contamination of the examinees' clothes and the operator's protective equipment. In addition, it can improve the examination experience and satisfaction of the examinees.

There are also certain limitations in this study. First, the sample size of the study is insufficient, and the subsequent study needs to increase the sample size to obtain more representative data. Second, there is a lack of statistics on the success rate of microscopic examination, and more rigorous evaluation indicators will be designed in the subsequent study to improve the scientific content of the study.

## Conclusion

The homemade modified colonoscopy nursing pad designed in this study did not affect the doctor to put the enteroscope into anus during the application, protected the privacy of the examinees, and solved the problem of contaminating the bed unit and clothing during the treatment of the examinees. At the end of the examination, the extension could be used to clean the perianal area and buttocks, and finally the whole used nursing pad was wrapped and discarded to avoid cross infection. This homemade modified colonoscopy nursing pad has important clinical significance and practical value, and is worthy of clinical promotion and use.

## Supplementary Information

**Additional file 1.** Satisfaction Questionnaire Form.

## Data Availability

All data generated or analyzed during this study are included in this article.
